# The association between socioeconomic position and vigorous physical activity among adolescents: a cross-sectional study in six European cities

**DOI:** 10.1186/s12889-021-10791-z

**Published:** 2021-05-05

**Authors:** L. Falese, B. Federico, A. E. Kunst, J. Perelman, M. Richter, A. Rimpelä, V. Lorant

**Affiliations:** 1 Department of Human Sciences, Society and Health, University of Cassino and Southern Lazio, via S. Angelo (Folcara), 03043 Cassino, FR Italy; 2 Department of Public Health, Amsterdam UMC, University of Amsterdam, Amsterdam, The Netherlands; 3 NOVA National School of Public Health, Public Health Research Centre, Universidade NOVA de Lisboa, Lisbon, Portugal; 4 Institute of Medical Sociology, Medical Faculty, Martin Luther University Halle-Wittenberg, Halle (Saale), Germany; 5 Faculty of Social Sciences, Unit of Health Sciences, Tampere University, Tampere, Finland; 6 Department of Adolescent Psychiatry, Tampere University Hospital, Nokia, Finland; 7 Institute of Health and Society, Université Catholique de Louvain, Bruxelles, Belgium

**Keywords:** Socioeconomic position, Vigorous physical activity, Adolescents

## Abstract

**Background:**

The relationship between socioeconomic position (SEP) and adolescent physical activity is uncertain, as most evidence is limited to specific settings and a restricted number of SEP indicators. This study aimed to assess the magnitude of socioeconomic differences in adolescent vigorous physical activity (VPA) across various European countries using a wide range of SEP indicators, including family-based (education, family affluence, perceived social standing, parents’ employment, housing tenure) and adolescent-based (academic performance and pocket money) ones.

**Methods:**

We used data from a survey among 10,510 students aged 14–17 from 50 schools in six European cities: Namur (BE), Tampere (FI), Hannover (DE), Latina (IT), Amersfoort (NL), Coimbra (PT). The questionnaire included socio-demographic characteristics and the amount of time spent in VPA.

**Results:**

The mean time spent practicing VPA was 60.4 min per day, with lower values for Namur (BE) and Latina (IT), and higher values for Amersfoort (NL). In the multivariable analysis, both categories of SEP indicators (family-based and adolescent based indicators) were independently associated with VPA. For each SEP indicator, lower levels of VPA were recorded in lower socioeconomic groups. In the total sample, each additional category of low SEP was associated with a decrease in mean VPA of about 4 min per day.

**Conclusions:**

This study showed that across European cities adolescent VPA is positively related to both family-based SEP and adolescents’ own SEP. When analysing socioeconomic differences in adolescent VPA, one should consider the use of multiple indicators of SEP.

**Supplementary Information:**

The online version contains supplementary material available at 10.1186/s12889-021-10791-z.

## Background

Physical activity during adolescence has important implications for the prevention of chronic diseases [1] and it provides social and psychological benefits [2]. Physical activity can be classified according to its intensity (expressed in terms of energy expenditure) in light, moderate and vigorous [3]. In youth, vigorous physical activity (VPA) is likely to produce better health outcomes compared to moderate physical activity [4]. The World Health Organization (WHO) recommends that adolescents achieve at least an average of 60 min of moderate to vigorous physical activity each day and incorporate VPA, as well as activities that strengthen muscle and bone such as playing games, running, jumping, at least three times a week [3]. However, most European adolescents, especially those in southern Europe, do not seem to meet these recommendations [5, 6].

An important predictor of health behaviours in young people is Socio Economic Position (SEP), which can be measured using both family-based and adolescent-based indicators [7]. The relationship between low SEP and unhealthy behaviours such as tobacco and alcohol abuse has been previously examined [8–10], but the association between low SEP and physical activity is less clear. A previous study that used family-based indicators of SEP showed that ownership of a house, an indicator of financial position, was positively associated with VPA [11] but this result was not confirmed in another study [12]. Family affluence was positively correlated with physical activity in the 32 countries participating in the Health Behaviour in School-aged Children survey [13], but the relationship was not significant in a previous study [14].

During adolescence, individuals develop their own social capital and may have financial resources at their disposal [7, 15]. Therefore, adolescent own SEP, in addition to family SEP, may have an important role in health behaviours. Some studies found higher individual disposable income to be associated with unhealthy behaviours (e.g. alcohol and tobacco consumption) [10, 16]. However, this SEP indicator has not been studied in relationship to physical activity so far. Academic performance, which can be considered as another indicator of adolescent individual SEP [10], was positively associated with physical activity in previous studies [17].

Previous research on the relationship between adolescents’ physical activity and SEP mainly relied either on one or two indicators of SEP, which may be inadequate to reveal socioeconomic differences in adolescents’ physical activity [18]. The observed differences in study results may in addition derive from differences in national contexts and in the measurement of physical activity [19, 20].

Little research has been carried out so far on socio economic differences in VPA even though team sports, soccer, running and athletics (all activities which are classified as vigorous by the WHO) are the most commonly reported physical activities among adolescents [21]. It is possible that socioeconomic differences in physical activity mainly derive from participation in sports, which imply greater energy expenditure than moderate-to-vigorous physical activity [22], and may be hampered by economic limitations and other barriers [23].

Therefore, the aim of this study was to measure the extent of socioeconomic differences in adolescent VPA relying on both family-based and adolescent own indicators of SEP. A further aim of this study was to measure if the strength of the association between SEP and adolescents’ VPA differs across several European cities.

## Methods

Data were collected from 11,015 students in 50 schools participating in the European SILNE study (Tackling socio-economic inequalities in smoking: learning from natural experiments by time trend analyses and cross-national comparisons) in 6 European cities having a similar social structure (e.g. unemployment, income, migration) compared to their country average: Namur (Belgium), Latina (Italy), Tampere (Finland), Hannover (Germany), Amersfoort (Netherlands) and Coimbra (Portugal). In each city, a sample of 6 to 8 schools was selected, and all students from two selected grades in secondary school were invited to complete the survey during school hours. The participation rate was 79.4%. By means of a self-completed questionnaire, we collected information on gender, age, health and lifestyles, family and individual SEP. The survey protocol and design is described in detail in a previous paper by Lorant et al. (2015) [9]. We included in the analysis only students aged 14–17 (*N* = 10,510).

### Measurement of vigorous physical activity (VPA)

Vigorous physical activity was measured using a composite question validated by Wong et al. [24] and used in the Youth Smokey Survey of Canada questionnaire [25]. The question was: “Mark how many minutes of vigorous physical activity you did on each of the last 7 days. This includes physical activity during physical education class, lunch, recess, after school, evening, and spare time”.

A definition of VPA that takes into consideration the WHO’s definition of VPA according to the amount of Metabolic Equivalent of task or METS performed [3], was given in the questionnaire: “Vigorous physical activities are considered as the ones that increase heart rate and make you breathe hard and sweat such as jogging, cycling, team sports, fast dancing, jump-rope”.

Respondents selected the possible answers (0, 30 min, 1 h, 1 h and a half, 2 h, 2 h and a half, 3 h or more) for each day of a typical week. For each participant, the time spent in VPA was summed up for each day of the week, and then divided by 7, thus obtaining the average time (minutes) engaged in VPA each day. Respondents who answered “3 hours or more” (*N* = 600, which is about 6% of total participants) were assigned 3 h in the calculation.

### Measurement of socioeconomic variables

#### Family-based indicators of SEP

Information on each parent’s educational level was provided by the question “What was the highest level of schooling your mother/father completed?” We used data from the parent with the highest level of education. The educational distribution of either parent was then grouped into 3 classes (low, middle, high). In order to keep in the analysis subjects with missing data for education, an additional category (“level of education unknown”) was created. Parent employment status was assessed by the question “Did your father/mother work during the last two weeks?” Possible answer were yes/no/I don’t know. The Family Affluence Scale (FAS) was used [13, 26, 27], which included the following items: Does your family own a car, van or truck? (0 No, 1 Yes, one, 2 Yes, two or more); Do you have your own bedroom for yourself? (0 No, 1 Yes); During the past 12 months, how many times did you go on holiday with your family? (0 Not at all, 1 Once, 2 Twice, 3 More than twice); How many computers does your family own? (0 None, 1 One, 2 Two, 3 More than two). A composite FAS score was calculated for each student summing these items. The two highest response categories (“2 “and “3 or more”) of the last two items (holidays and computers) were combined, thus obtaining values of FAS ranging from 0 to 7 [26]. After calculation of the FAS score, city-specific tertiles were computed and three levels of FAS were created (low, middle, high). The MacArthur Scale assesses students’ perception of their families’ social standing according to a rank which goes from 0 to 10 [28]. Values were re-coded into city-specific tertiles for the analysis. Respondents reported whether or not their parents owned the house.

#### Adolescent own indicators of SEP

Disposable income was assessed by the question “How much money do you usually get each week to spend for yourself or to save as pocket money?” [29]. Data were divided in three categories: 0–5; 6–20; more than 20 euro per week. Adolescent academic achievement score was obtained from the student’s marks during the previous school year (classified in five categories: insufficient, low, average, good or high achievement) [10]. Data were then recoded in three categories: low, middle and high.

#### Summary SEP variable

In order to evaluate whether SEP differences varied by city, we created a summary SEP variable based on the number of times an adolescent was in the lowest SEP category [30]. This variable is a count of the number of lowest SEP categories each student belongs to. A greater number corresponds to more disadvantage: the greater the number of low SEP categories, the lower the SEP.

### Statistical analysis

A multilevel multivariable linear regression model that used VPA as dependent variable was built, using fixed effects for cities and random effects for schools, respectively. The analysis was performed in two steps: firstly, a multivariable model with all SEP indicators as well as age, sex and city dummies as covariates was built. Subjects with missing values in one or more covariates (with the exception of parental education) were excluded from this analysis: the multivariable regression model was thus performed on 9722 adolescents. Deviation-from-means coding was used for city dummy variables, so each city coefficient represent the difference between the city and the average of the six cities. Secondly, we fitted a multivariable model with interaction terms between city dummies and SEP. This latter model included a new indicator of SEP, that represents the number of lowest SEP categories each student belonged to [30]. In the regression model with interaction terms, the country-specific regression coefficients represent departures from the “average slope”.

All statistical analyses were performed using the software package Stata 14.

## Results

Table 1 shows participants’ characteristics and average daily VPA for each category of the independent variables. In the whole sample, the average time spent in VPA was 60.4 min per day; higher mean values were found in Amersfoort (NL) with an average of 80.7 min per day followed by Tampere (FI), whereas Latina (IT) and Namur (BE) had the lowest mean values. The percentage of missing data was very small for all variables, with the exception of parental education (12.5% missing). Table 1 shows also crude and adjusted differences in VPA for each indicator of SEP, with 95% Confidence Intervals. For all SEP indicators, differences in VPA were smaller in the multivariable analysis than at the univariable analysis, but all remained significant at the 0.05 level, except parental occupation. Both categories of SEP indicators (family-based and adolescent own indicators) were independently associated with VPA in the multivariable analysis: the higher the SEP, the greater the time spent in VPA. Furthermore, all indicators of SEP measured on an ordinal scale (parental education, family affluence, perceived social standing, personal income and academic achievement) showed a dose-response relationship with VPA. When the analysis was stratified by city, the indicators of SEP played a different role according to the city considered, but the presence of socioeconomic inequalities in VPA emerged in all cities (Appendix 1).

**Table 1:** Characteristics of the sample and results of the multilevel multivariable linear regression model for Vigorous Physical Activity (minutes per day)

		N	Mean Vigorous Physical Activity (minutes per day)	Crude difference	Adjusted difference^a^	95% Confidence Interval for the adjusted difference
Sex	Female	5476	52.9	Ref.	Ref.	
	Male	5001	68.7	15.8	14.6	(13.2, 16.0)
	Not available	33				
Age (years)	14-15	6833	62.6	Ref.	Ref.	
	16-17	3677	56.3	-6.3	-1.6	(-3.2, -0.0)
	Not available	0				
Parental education	Low	1269	53.5	Ref.	Ref.	
	Middle	3706	57.8	4.3	2.0	(-0.4, 4.3)
	High	4226	64.8	11.3	2.7	(0.3, 5.1)
	Not available	1309				
Family Affluence Scale	I tertile	3350	55.6	Ref.	Ref.	
	II tertile	3060	60.2	4.6	1.2	(-0.6, 3.0)
	III tertile	4100	64.5	8.9	4.7	(2.8, 6.6)
	Not available	0				
MacArthur Scale	I tertile	3085	54.3	Ref.	Ref.	
	II tertile	3925	59.7	5.4	1.3	(-0.4, 3.1)
	III tertile	3215	67.3	13.0	4.4	(2.5, 6.3)
	Not available	308				
Parental employment	One or both employed	9875	61.0	Ref.	Ref.	
	Both not in employment	635	50.9	-10.1	-2.5	(-5.6, 0.6)
	Not available	0				
Housing tenure	No	1875	54.7	Ref.	Ref.	
	Yes	8480	61.7	7.0	2.8	(0.9, 4.7)
	Not available	155				
Weekly personal income	0-5 euro	2409	53.6	Ref.	Ref.	
	6-20 euro	4907	59.9	6.3	2.0	(0.2, 3.7)
	>20 euro	2996	67.3	13.7	4.7	(2.7, 6.8)
	Not available	198				
Academic achievement	Low	1935	57.4	Ref.	Ref.	
	Middle	4430	61.2	3.8	2.3	(0.3, 4.3)
	High	4298	61.2	3.8	4.5	(2.5, 6.6)
	Not available	281				
City^b^	Namur (BE)	2002	50.8	-9.6	-11.5	(-14.6, -8.4)
	Tampere (FI)	1492	66.8	6.4	6.1	(3.0, 9.2)
	Hannover (GE)	1348	61.9	1.5	2.2	(-0.7, 5.1)
	Latina (IT)	2045	50.8	-9.6	-8.6	(-11.7, -5.6)
	Amersfoort (NL)	1870	80.7	20.3	17.7	(14.7, 20.8)
	Coimbra (PT)	1753	54.1	-6.3	-5.9	(-9.3, -2.5)

^a^Coefficient of the multilevel multivariable linear regression model (minutes per day)

^b^Deviation from means coding was used in the regression model, thus the reference group is the mean of all cities

Finally, a summary SEP variable, which represents the number of lowest SEP categories each student belonged to, was factored in solely and interacted with city. The frequency distribution of this summary SEP variable is shown in the Appendix 2 for each city.

Table 2 shows the mean values of VPA for each value of the summary SEP variable, as well as the regression coefficients of the linear regression model of VPA on SEP. The average slope of the regression line was − 4.1 (95% CI -4.7, − 3.5), which implies that each additional category of low SEP is associated with a decrease in mean VPA of about 4 min per day. The slope of the regression line was negative in all 6 European cities under investigation, being less marked in Coimbra (PT) and more marked in Tampere (FI).

**Table 2:** Mean values of Vigorous Physical Activity (VPA) by number of categories of low Socio Economic Position (SEP) and city and results of the linear regression model for VPA with interactions between low SEP and city

	Mean VPA (minutes per day) by number of low SEP categories				Coefficients of the linear regression of low SEP on VPA^a^			
	0	1	2	≥3	Intercept (95% CI)		Slope (95% CI)	
Namur (BE)	55.2	53.4	49.2	41.7	−11.9	(−15.5, −8.3)	0.7	(−0.6, 2.0)
Tampere (FI)	76.6	66.5	63.2	54.1	9.8	(6.2, 13.5)	−3.3	(−4.8, −1.9)
Hannover (GE)	69.6	63.6	55.0	57.1	2.1	(−1.6, 5.7)	−0.6	(−2.1, 1.0)
Latina (IT)	55.9	51.6	50.6	42.4	−10.2	(− 13.7, −6.6)	1.0	(−0.3, 2.3)
Amersfoort (NL)	83.0	81.2	80.4	70.4	17.4	(13.9, 20.8)	0.7	(−0.7, 2.2)
Coimbra (PT)	58.1	56.1	51.2	50.5	−7.2	(−11.1, −3.3)	1.4	(0.1, 2.8)
*Total*	*67.3*	*61.8*	*57.0*	*50.5*	*59.3*	*(57.5, 61.1)*	*−4.1*	*(−4.7, −3.5)*

*VPA* Vigorous Physical Activity, *SEP* Socio Economic Position

^a^Deviation from means coding was used in the regression model, thus the reference group is the mean of all cities

## Discussion

This study showed that VPA is less prevalent in lower socioeconomic groups. In the multivariable analysis, both family-based SEP indicators and adolescent own SEP indicators were independently associated with VPA. Furthermore, socioeconomic differences in VPA were found in all 6 European cities under investigation, and they were more evident in Tampere (FI) and less marked in Coimbra (PT).

Socioeconomic differences in VPA can emerge through multiple causal pathways [31]. Parental education indicates the level of knowledge of the parents, which can include knowledge of the health benefits provided by the regular practice of physical activity [32]. Higher educated parents can thus be more inclined to support their children’s engagement in physical activity or choose schools that promote physical activity [32]. On the other hand, families with lower financial resources may find it difficult to access sport infrastructures, pay for club membership fees, or practice sports that require expensive equipment [32, 33]. Currently, an important share of youth physical activity takes place in organised settings [5, 34], which can enhance socioeconomic differences in physical activity. A further explanation of the observed SEP differences in VPA relates to psychological mechanisms, which are especially important during the critical developmental period of adolescence [35]: living in disadvantaged socioeconomic positions may be linked to greater stress, anxiety and depression, which in turn can discourage adolescents to engage in physical activity [36–38].

In this study, personal SEP was significantly associated with VPA even after adjustment for family SEP: adolescents with a greater availability of pocket money and better academic performance were more physically active than those with little or no pocket money. In previous studies, success in school was found to be associated with higher self-esteem and psychological adjustment, which in turn show positive association with physical activity [39, 40]. Adolescent physical activity can therefore be considered a “normal good”, whose consumption increases with income, likewise unhealthy behaviours such as eating chips [41].

This study has some strength and a few limitations. The main strengths are the cross-comparative nature of the survey based on a large sample size and the possibility to apply the same research methods across 6 different European cities. Other strengths include the high participation rate (79%) and the analysis of many different indicators for SEP, including indicators based on family characteristics as well as indicators of adolescent attributes.

An important limitation of this study relates to the convenience sample: as described in the Methods section, data were collected in those schools that agreed to participated in a European study on smoking involving only one city for each of 6 countries. The choice of the sample was influenced by both statistical and opportunity reasons: as a consequence, study results can neither be considered representative of the 6 countries, nor of the 6 cities examined. Nonetheless, we found consistent associations between VPA and SEP throughout the 6 cities. A second limitation of the study relates to the validity of self-reported data. In particular, self-reports of physical activity intensity could produce either overestimation or underestimation [42]. It is possible that the concept of VPA itself was misinterpreted, even though the questionnaire included the definition of VPA. A previous study on European adolescents reported significantly lower mean values of VPA, when objectively assessed by means of an accelerometer, compared to the current study [43]. Participants may thus have included time spent in moderate physical activity, and not only VPA as requested. It was also found that higher SEP adolescents may be more inclined to report intense physical activity than lower SEP adolescents due to social desirability bias [22]. However, it is unlikely that these social desirability bias accounts for the observed differences across all the SEP indicators examined.

In conclusion, the results of this study contribute to a better mapping of socioeconomic differences in adolescent VPA. We recommend that future studies use multiple indicators of SEP and consider the use of adolescent own indicators of SEP.

## Supplementary Information

**Additional file 1: Appendix 1**. Results of the multilevel linear regression model for Vigorous Physical Activity (minutes per day) with stratification by city. **Appendix 2**. Percentage distribution of the number of low Socio Economic Position (SEP) categories by city.

## Data Availability

The datasets used and/or analysed during the current study are available from the corresponding author on reasonable request.

## Abbreviations

SEP: Socioeconomic position
VPA: Vigorous physical activity
WHO: The World Health Organization
FAS: The Family Affluence Scale

## References

[1] Loprinzi PD, Cardinal BJ, Loprinzi KL, Lee H (2012). Benefits and environmental determinants of physical activity in children and adolescents. Obes Facts.

[2] Eime RM, Young JA, Harvey JT, Charity MJ, Payne WR (2013). A systematic review of the psychological and social benefits of participation in sport for adults: informing development of a conceptual model of health through sport. Int J Behav Nutr Phys Act.

[3] WHO (2020). Guidelines on physical activity and sedentary behaviour.

[4] Owens S, Galloway R, Gutin B (2017). The case for vigorous physical activity in youth. Am J Lifestyle Med.

[5] European Commission (2017). Special Eurobarometer 472 “sport and physical activity”.

[6] WHO (2018). Physical Activity Factsheets for the 28 European Union Member States of the WHO European Region: World Health Organization.

[7] Moor I, Kuipers MAG, Lorant V, Pförtner TK, Kinnunen JM, Rathmann K, et al. Inequalities in adolescent self-rated health and smoking in Europe: comparing different indicators of socioeconomic status. J Epidemiol Community Health. 2019;73(10):963–70. 10.1136/jech-2018-211794. 10.1136/jech-2018-211794 31302594

[8] Kuipers MAG, Monshouwer K, van Laar M, Kunst AE (2015). Tobacco control and socioeconomic inequalities in adolescent smoking in Europe. Am J Prev Med.

[9] Lorant V, Soto VE, Alves J, Federico B, Kinnunen J, Kuipers M (2015). Smoking in school-aged adolescents: design of a social network survey in six European countries. BMC Res Notes.

[10] Bosque-Prous M, Kuipers MAG, Espelt A, Richter M, Rimpela A, Perelman J (2017). Adolescent alcohol use and parental and adolescent socioeconomic position in six European cities. BMC Public Health.

[11] Utter J, Denny S, Robinson EM, Ameratunga S, Watson P (2006). Perceived access to community facilities, social motivation, and physical activity among New Zealand youth. J Adolesc Health.

[12] Shi Z, Lien N, Kumar BN, Holmboe-Ottesen G (2006). Physical activity and associated socio-demographic factors among school adolescents in Jiangsu Province, China. Prev Med.

[13] Borraccino A, Lemma P, Iannotti RJ, Zambon A, Dalmasso P, Lazzeri G (2009). Socioeconomic effects on meeting physical activity guidelines: comparisons among 32 countries. Med Sci Sports Exerc.

[14] Haug E, Torsheim T, Samdal O (2008). Physical environmental characteristics and individual interests as correlates of physical activity in Norwegian secondary schools: the health behaviour in school-aged children study. Int J Behav Nutr Phys Act.

[15] Steinberg L, Morris AS (2001). Adolescent development. Annu Rev Psychol.

[16] Perelman J, Alves J, Pfoertner TK, Moor I, Federico B, Kuipers MAG, et al. The association between personal income and smoking among adolescents: a study in six European cities. Addiction. 2017;112(12):2248–56. 10.1111/add.13930. 10.1111/add.13930 PMC5698771 28667824

[17] Álvarez-Bueno C, Pesce C, Cavero-Redondo I, Sánchez-López M, Garrido-Miguel M, Martínez-Vizcaíno V. Academic achievement and physical activity: a meta-analysis. Pediatrics. 2017;140(6). 10.1542/peds.2017-1498 29175972

[18] Braveman PA, Cubbin C, Egerter S, Chideya S, Marchi KS, Metzler M, et al. Socioeconomic status in health research: one size does not fit all. JAMA. 2005;294(22):2879–88. 10.1001/jama.294.22.2879. 10.1001/jama.294.22.2879 16352796

[19] Langlois J, Omorou AY, Vuillemin A, Briançon S, Lecomte E, Group PT (2017). Association of socioeconomic, school-related and family factors and physical activity and sedentary behaviour among adolescents: multilevel analysis of the PRALIMAP trial inclusion data. BMC Public Health.

[20] Stalsberg R, Pedersen AV. Are differences in physical activity across socioeconomic groups associated with choice of physical activity variables to report? Int J Environ Res Public Health. 2018;15(5). 10.3390/ijerph15050922 PMC5981961 29734745

[21] Hulteen RM, Smith JJ, Morgan PJ, Barnett LM, Hallal PC, Colyvas K, et al. Global participation in sport and leisure-time physical activities: a systematic review and meta-analysis. Prev Med. 2017;95:14–25. 10.1016/j.ypmed.2016.11.027. 10.1016/j.ypmed.2016.11.027 27939265

[22] Maher CA, Olds TS (2011). Minutes, MET minutes, and METs: unpacking socio-economic gradients in physical activity in adolescents. J Epidemiol Community Health.

[23] Eime RM, Harvey JT, Craike MJ, Symons CM, Payne WR (2013). Family support and ease of access link socio-economic status and sports club membership in adolescent girls: a mediation study. Int J Behav Nutr Phys Act.

[24] Wong SL, Leatherdale ST, Manske SR (2006). Reliability and validity of a school-based physical activity questionnaire. Med Sci Sports Exerc.

[25] Kroeker C, Manske S, Rynard V, Smith E (2012). Validity and reliability of the measures for youth respondents from the core indicators and measures of youth health tobacco control and physical activity & sedentary behaviour modules.

[26] Currie C, Molcho M, Boyce W, Holstein B, Torsheim T, Richter M (2008). Researching health inequalities in adolescents: the development of the health behaviour in school-aged children (HBSC) family affluence scale. Soc Sci Med.

[27] Torsheim T, Cavallo F, Levin KA, Schnohr C, Mazur J, Niclasen B (2016). Psychometric validation of the revised family affluence scale: a latent variable approach. Child Indic Res.

[28] Goodman E, Adler NE, Kawachi I, Frazier AL, Huang B, Colditz GA (2001). Adolescents' perceptions of social status: development and evaluation of a new indicator. Pediatrics..

[29] Scragg R, Laugesen M, Robinson E (2002). Cigarette smoking, pocket money and socioeconomic status: results from a national survey of 4th form students in 2000. N Z Med J.

[30] Lorant V, Rojas VS, Robert PO, Kinnunen JM, Kuipers MA, Moor I (2017). Social network and inequalities in smoking amongst school-aged adolescents in six European countries. Int J Public Health.

[31] Jo Inchley DC, Young T, Oddrun Samdal TT, Augustson L, Frida Mathison AA-D, Molcho M, Barnekow MWV, WHO (2016). Growing up unequal: gender and socioeconomic differences in young people’s health and well-being. Health Behaviour In School-Aged Children (Hbsc) Study: International Report From The 2013/2014 Survey.

[32] Federico B, Falese L, Capelli G (2009). Socio-economic inequalities in physical activity practice among Italian children and adolescents: a cross-sectional study. J Sports Sci.

[33] Higgerson J, Halliday E, Ortiz-Nunez A, Brown R, Barr B (2018). Impact of free access to leisure facilities and community outreach on inequalities in physical activity: a quasi-experimental study. J Epidemiol Community Health.

[34] Jekauc D, Reimers AK, Wagner MO, Woll A (2013). Physical activity in sport clubs of children and adolescents in Germany: results from a nationwide representative survey. J Public Health.

[35] Cundiff JM, Matthews KA (2017). Is subjective social status a unique correlate of physical health? A meta-analysis. Health Psychol.

[36] Ng DM, Jeffery RW (2003). Relationships between perceived stress and health behaviors in a sample of working adults. Health Psychol.

[37] Gerber M, Kalak N, Lemola S, Clough PJ, Pühse U, Elliot C, et al. Adolescents' exercise and physical activity are associated with mental toughness. Ment Health Phys Act. 2012;5(1):35–42. 10.1016/j.mhpa.2012.02.004.

[38] Quon EC, McGrath JJ (2014). Subjective socioeconomic status and adolescent health: a meta-analysis. Health Psychol.

[39] Zamani Sani SH, Fathirezaie Z, Brand S, Pühse U, Holsboer-Trachsler E, Gerber M, et al. Physical activity and self-esteem: testing direct and indirect relationships associated with psychological and physical mechanisms. Neuropsychiatr Dis Treat. 2016;12:2617–25. 10.2147/NDT.S116811. 10.2147/NDT.S116811 PMC5068479 27789950

[40] Tremblay MS, Inman J, Willms J (2000). The relationship between physical activity, self-esteem, and academic achievement in 12-year-old children. Pediatr Exerc Sci.

[41] Currie CE, Elton RA, Todd J, Platt S (1997). Indicators of socioeconomic status for adolescents: the WHO health behaviour in school-aged children survey. Health Educ Res.

[42] McCormack G, Giles-Corti B, Lange A, Smith T, Martin K, Pikora TJ (2004). An update of recent evidence of the relationship between objective and self-report measures of the physical environment and physical activity behaviours. J Sci Med Sport.

[43] Jiménez-Pavón D, Fernández-Vázquez A, Alexy U, Pedrero R, Cuenca-García M, Polito A, et al. Association of objectively measured physical activity with body components in European adolescents. BMC Public Health. 2013;13(1):667. 10.1186/1471-2458-13-667. 10.1186/1471-2458-13-667 PMC3723445 23866681

