# Supplementary material for: The association between socioeconomic position and vigorous physical activity among adolescents: a cross-sectional study in six European cities
Source: BMC Public Health. 2021 May 5;21:866. doi: 10.1186/s12889-021-10791-z (PMC8097935; doi:10.1186/s12889-021-10791-z)
Supplement: Supplementary file 1 — Additional file 1: Appendix 1. Results of the multilevel linear regression model for Vigorous Physical Activity (minutes per day) with stratification by city. Appendix 2. Percentage distribution of the number of low Socio Economic Position (SEP) categories by city. [file 12889_2021_10791_MOESM1_ESM.docx]

Appendix 1. Results of the multilevel linear regression model for Vigorous Physical Activity (minutes per day) with stratification by city

|  |  | Namur (BE) | Tampere (FI) | Hannover (GE) | Latina (IT) | Amersfoort (NL) | Coimbra (PT) |
| --- | --- | --- | --- | --- | --- | --- | --- |
|  |  | N=1824 | N=1386 | N=1229 | N=1878 | N=1801 | N=1604 |
|  |  |  |  |  |  |  |  |
| Parental education | Low | Ref. | Ref. | Ref. | Ref. | Ref. | Ref. |
|  | Middle | 3.4 | 3.3 | -2.1 | 2.0 | 3.5 | 3.2 |
|  | High | 4.6 | 1.5 | -2.1 | **5.4** | 1.6 | **4.7** |
|  |  |  |  |  |  |  |  |
| Family Affluence Scale | I TERTILE | Ref. | Ref. | Ref. | Ref. | Ref. | Ref. |
|  | II TERTILE | 0.1 | 1.3 | 0.0 | 0.5 | 1.9 | 1.9 |
|  | III TERTILE | **6.6** | **6.9** | 1.9 | **5.9** | 0.7 | 3.4 |
|  |  |  |  |  |  |  |  |
| MacArthur Scale | I TERTILE | Ref. | Ref. | Ref. | Ref. | Ref. | Ref. |
|  | II TERTILE | 0.1 | 5.2 | 3.4 | -0.1 | 1.5 | -0.4 |
|  | III TERTILE | 1.7 | **10.7** | **9.2** | 3.2 | **5.3** | -0.2 |
|  |  |  |  |  |  |  |  |
| Parental employment | One or both employed | Ref. | Ref. | Ref. | Ref. | Ref. | Ref. |
|  | Both not in employment | -1.9 | 2.3 | -2.4 | 0.4 | -9.2 | -4.0 |
|  |  |  |  |  |  |  |  |
| Housing tenure | No | Ref. | Ref. | Ref. | Ref. | Ref. | Ref. |
|  | Yes | 1.1 | **6.1** | 4.7 | 0.4 | 1.5 | -0.9 |
|  |  |  |  |  |  |  |  |
| Weekly personal income | 0-5 euro | Ref. | Ref. | Ref. | Ref. | Ref. | Ref. |
|  | 6-20 euro | 3.1 | -0.2 | 2.0 | 1.8 | 5.6 | 1.6 |
|  | >20 euro | 2.3 | 3.4 | **6.1** | 3.1 | **10.4** | 4.5 |
|  |  |  |  |  |  |  |  |
| Academic achievement | Low | Ref. | Ref. | Ref. | Ref. | Ref. | Ref. |
|  | Middle | 4.7 | **5.8** | 2.1 | 2.7 | -0.0 | -1.7 |
|  | High | 1.9 | **7.8** | 2.3 | **7.2** | 2.9 | **5.0** |

Coefficients are age and sex-adjusted. Significant differences (p<0.05) are shown in **bold**

Appendix 2. Percentage distribution of the number of low Socio Economic Position (SEP) categories by city

|  | **Percentage distribution** | | | |
| --- | --- | --- | --- | --- |
|  | **of the number of low SEP categories** | | | |
|  | **0** | **1** | **2** | **≥3** |
| Namur (BE) | *29.1* | *30.2* | *21.0* | *19.7* |
| Tampere (FI) | *31.4* | *30.9* | *19.2* | *18.4* |
| Hannover (GE) | *27.9* | *27.5* | *21.6* | *23.1* |
| Latina (IT) | *28.3* | *31.3* | *21.3* | *19.1* |
| Amersfoort (NL) | *42.2* | *31.6* | *16.3* | *10.0* |
| Coimbra (PT) | *25.7* | *26.0* | *23.5* | *24.8* |
| *Total* | *30.9* | *29.7* | *20.5* | *18.9* |

SEP = Socio-Economic Position
